# Cochlear radiation dose and hearing loss in patients with vestibular schwannoma undergoing radiosurgery: systematic review^☆^

**DOI:** 10.1016/j.bjorl.2023.101300

**Published:** 2023-07-19

**Authors:** Gabriela Silva Teixeira Cavalcanti, Andrea Lemos, Eduarda C. Moretti, Camilla Maria G.A. Lucena, João Gabriel R. Gomes, Lílian F. Muniz, Leonardo G.A. Venâncio, Silvio Caldas, Mariana C. Leal

**Affiliations:** a Universidade Federal de Pernambuco, Centro de Ciências da Saúde, Recife, PE, Brazil; b Universidade Federal de Alagoas, Instituto de Ciências Biológicas e da Saúde, Maceió, AL, Brazil; c Real Hospital Português, Departamento de Neurocirurgia, Recife, PE, Brazil; d Universidade Federal de Pernambuco, Centro de Ciências Médicas, Recife, PE, Brazil

**Keywords:** Acoustic neuroma, Hearing loss, Radiation dose, Radiosurgery, Systematic review

## Abstract

**Objectives:**

To determine the cut-off point of the cochlear radiation dose as a risk factor for hearing loss in patients with vestibular schwannoma treated with radiosurgery.

**Methods:**

A systematic review of the literature was performed without language or publication year restrictions in the MEDLINE/PubMed, EMBASE, Web of Science, LILACS/VHL and Cochrane Library databases. Studies that met the following criteria were included: 1) population: adults of both sexes who underwent radiosurgery for vestibular schwannoma treatment; 2) exposure: cochlear radiation; 3) outcome: hearing loss; 4) type of study: cohort. Two independent reviewers conducted the entire review process. The registration number in PROSPERO was CRD42020206128.

**Results:**

From the 333 articles identified in the searches, seven were included after applying the eligibility criteria. There was no standardization as to how to measure exposure or outcome in the included studies, and most studies did not present sufficient data to enable meta-analysis.

**Conclusion:**

It was not possible to determine a cut-off point for high cochlear dose that could be considered a risk factor for hearing loss.

## Highlights

• The authors are unaware of a similar review until the search date.
• Cochlear dose measurement method varied between studies.
• The criterion to define the decline in hearing function also differed between studies.
• Publication bias was noted in the cohorts due to incomplete presentation of data.

## Introduction

Vestibular schwannoma treatment aims to slow tumor growth and avoid the sequelae caused possibly due to the compressive effect on noble anatomical structures of the internal auditory canal and the cerebellopontine angle cistern.^1^ Therapeutic options include expectant management with imaging, conventional radiation therapy, Stereotactic Radiosurgery (SRS), or microsurgery follow-up.^1^, ^2^ The decision of the best modality should be individualized, considering the patient’s psychosocial factors, tumor characteristics, symptomatology, and the healthcare staff’s expertise.^3^, ^4^

Amongst the therapeutic possibilities, SRS has shown excellent results in preventing tumor progression. It consists of administering a single 11–16 gray (Gy) radiation dose, which should be spread across the entire tumor surface. Although minimally invasive and designed to provide conformational radiation with a marked downward dose gradient curve to the periphery, SRS can yet expose structures adjacent to the tumor to intolerable radiation doses.^5^, ^6^

Despite presenting a better rate of hearing preservation when compared to other therapeutic modalities, SRS can cause a progressive and late decline in auditory function with significant frequency, with cases of sudden hearing loss more rarely observed.^7^, ^8^, ^9^, ^10^ The exact mechanism of late hearing deterioration after SRS is not fully elucidated.^7^, ^11^ It is assumed that the cochlear radiation dose, which is an undesirable effect of SRS and consists of the amount of radiation deposited in the cochlea during radiosurgery, is a prognostic factor for hearing function, but studies still fail to prove this correlation.^6^, ^12^, ^13^, ^14^ There is no consensus regarding the dose limits used in the studies that found a positive correlation.^15^, ^16^, ^17^, ^18^, ^19^, ^20^, ^21^, ^22^, ^23^, ^24^, ^25^

Therefore, this review aims to determine the cut-off point of the cochlear radiation dose as a risk factor for declining hearing function in patients with vestibular schwannoma treated with radiosurgery.

## Methods

The protocol of this systematic review was registered and approved by PROSPERO in 2020 under the PROSPERO (CRD42020206128) registration number. The Meta-analysis of Observational Studies in Epidemiology (MOOSE) guidelines were used for producing and writing this review.^26^

### Search strategy

The search was conducted by an experienced librarian in January 2021 and updated in April 2022. The MEDLINE/PubMed, EMBASE, Web of Science, LILACS/VHL, Cochrane Library, and Brazilian Digital Library of Theses and Dissertations databases were used, with the descriptors shown in Table 1. Experts consulted for further article search.

**Table 1:** Search strategies for each database on the relationship between cochlear radiation dose and decline in auditory function in patients with vestibular schwannoma undergoing radiosurgery.

Database	Search strategy
MEDLINE	((“Neuroma, Acoustic”[mh] OR “VS”[tiab] OR “vss”[tiab] OR ((Acoustic[tiab] OR angle[tiab] OR vestibular[tiab]) AND Schwannoma*[tiab])) AND (Radiosurgery[mh] OR Radiosurger*[tiab] OR “radio-surgery”[tiab])) AND ((Constraint*[tiab] OR dose*[tiab] OR fraction*[tiab]) AND (Radiotherapy[mh] OR “Radiation Therapies”[tiab] OR “Radiation Therapy”[tiab] OR “Radiation Treatment”[tiab] OR “Radiation Treatments”[tiab] OR Radiotherap*[tiab] OR “Therapies, Radiation”[tiab] OR “Therapies, Targeted Radiation”[tiab] OR “Therapy, Radiation”[tiab] OR “Therapy, Targeted Radiation”[tiab] OR “Treatment, Radiation”[tiab]) AND Cochlea[mh] OR Cochlea*[tiab]) AND (Hearing[mh] OR “Hearing Disorders”[mh] OR “Auditory Perception”[mh] OR Hearing[tiab] OR audition[tiab] OR “Hearing, Distorted”[tiab] OR “Hearing Disorders”[tiab] OR “Distorted Hearing”[tiab] OR Dysacusis[tiab] OR “Hearing Disorder”[tiab] OR Paracousis[tiab] OR Paracusis[tiab] OR “Auditory Perceptions”[tiab] OR “Perception, Auditory”[tiab] OR “Perceptions, Auditory”[tiab] OR “Gardner–Robertson”[tiab] OR “GR”[tiab])
EMBASE	((‘acoustic neuroma’/exp OR ‘VS’:ti,ab OR ‘vss’:ti,ab OR ((Acoustic:ti,ab OR angle:ti,ab OR vestibular:ti,ab) AND Schwannoma*:ti,ab)) AND (Radiosurgery/exp OR Radiosurger*:ti,ab OR ‘radio-surgery’:ti,ab)) AND ((Constraint*:ti,ab OR dose*:ti,ab OR fraction*:ti,ab) AND (Radiotherapy/exp OR ‘Radiation Therapies’:ti,ab OR ‘Radiation Therapy’:ti,ab OR ‘Radiation Treatment’:ti,ab OR ‘Radiation Treatments’:ti,ab OR Radiotherap*:ti,ab OR ‘Therapies, Radiation’:ti,ab OR 'Therapies, Targeted Radiation':ti,ab OR 'Therapy, Radiation':ti,ab OR ‘Therapy, Targeted Radiation’:ti,ab OR ‘Treatment, Radiation’:ti,ab) AND Cochlea/exp OR Cochlea*:ti,ab) AND (Hearing/exp OR ‘Hearing Disorder’/exp OR ‘Auditory Perception’:ti,ab OR Hearing:ti,ab OR audition:ti,ab OR ‘Hearing, Distorted’:ti,ab OR ‘Hearing Disorders’:ti,ab OR ‘Distorted Hearing’:ti,ab OR Dysacusis:ti,ab OR ‘Hearing Disorder’:ti,ab OR Paracousis:ti,ab OR Paracusis:ti,ab OR ‘Auditory Perceptions’:ti,ab OR ‘Perception, Auditory’:ti,ab OR ‘Perceptions, Auditory’:ti,ab OR 'Gardner–Robertson':ti,ab OR 'GR':ti,ab)
WEB OF SCIENCE	ALL = (((“acoustic neuroma” OR “VS” OR “vss” OR ((Acoustic OR angle OR vestibular) AND Schwannoma*)) AND (Radiosurger* OR “radio-surgery” OR surger*)) AND ((Constraint* OR dose* OR fraction*) AND (“Radiation Therapies” OR “Radiation Therapy” OR “Radiation Treatment” OR “Radiation Treatments” OR Radiotherap* OR “Therapies, Radiation” OR “Therapies, Targeted Radiation” OR “Therapy, Radiation” OR “Therapy, Targeted Radiation” OR “Treatment, Radiation”) AND Cochlea*) AND (Hearing OR “Hearing Disorder” OR “Auditory Perception” OR Hearing OR audition OR “Hearing, Distorted” OR “Hearing Disorders” OR “Distorted Hearing” OR Dysacusis OR “Hearing Disorder” OR Paracousis OR Paracusis OR “Auditory Perceptions” OR “Perception, Auditory” OR “Perceptions, Auditory” OR “Gardner–Robertson” OR “GR”))
LILACS	(mh:”Neuroma Acústico” OR “Neuroma, Acoustic” OR “VS" OR “VSS” OR tw:(“Neuroma Acústico” OR “Neurinoma do Acústico” OR “Tumores do Ângulo Ponto-cerebelar” OR (Schwanoma* AND (vestibular OR acustico* OR Acoustic OR angle)))) AND (mh:Radiocirurgia OR tw:(Radiocirurgia OR Radiocirugia OR Radiosurger* OR “radio-surgery” OR radioterap* OR “Radiação Estereotáctica”)) AND (tw:(Constraint* OR dose* OR fraction* OR frac* OR dosag* OR dosi* OR Measurement OR medicao OR medicion OR “limite da radicao” OR limite)) AND (mh:Radiotherapy OR tw:(“Radiation Therapies” OR “Radiation Therapy” OR “Radiation Treatment” OR “Radiation Treatments” OR Radiotherap* OR “Therapies, Radiation” OR “Therapies, Targeted Radiation” OR “Therapy, Radiation” OR “Therapy, Targeted Radiation” OR “Treatment, Radiation” OR radioterapia OR “Tratamento por Radiação” OR “Tratamentos por Radiação” OR radiacion OR radiologico*)) AND (mh:Cochlea OR tw:Cochlea* OR modiolar* OR coclear*) AND (mh:(Hearing OR “Hearing Disorders” OR “Auditory Perception”) OR tw:(Hearing OR auditi* OR Dysacusis OR Paracousis OR Paracusis OR Auditory Perceptions” OR Auditory OR “Gardner–Robertson” OR “GR” OR audicao OR audicion))
BDTD	((“Neuroma, Acoustic” OR “VS” OR “vss” OR “Neuroma Acústico” OR “Neurinoma do Acústico” OR “Tumores do Ângulo Ponto-cerebelar”) OR (Acoustic OR angle OR vestibular OR acustico OR angle)) AND (Schwannoma OR Schwanoma)

Source: created by the author (2022).

Two independent reviewers evaluated the eligibility of the studies published by April 2022 without language or year of publication restrictions. Articles published in a language other than Portuguese or English were evaluated with the help of a translator. Disagreements were resolved by a third reviewer.

### Inclusion and exclusion criteria

Studies could be included if they met the following criteria: 1) population: adults (>20 years old) of both sexes that underwent radiosurgery for vestibular schwannoma treatment; 2) exposure: cochlea radiation; 3) outcome: decline of hearing function, quantified objectively with audiometric assessment; 4) type of study: prospective or retrospective cohort studies.

Studies containing samples of participants treated with hypofractionated radiosurgery or undergoing prior conventional radiotherapy or drug treatment to maintain auditory thresholds were excluded. Studies involving patients with anacusis before treatment or with neurofibromatosis type 2 were also excluded.

### Study selection

Study selection was performed with the Rayyan application, with which two independent reviewers analyzed the titles and abstracts of all studies identified from the search results. Studies that did not meet the eligibility criteria were excluded. The complete text of the remaining studies was retrieved and reevaluated by the two reviewers, also independently and using the same application. Studies that were not published could be included only if they presented the necessary data in the abstract. Disagreements between the two reviewers during the selection stage were resolved by a third evaluator. The studies in this stage were sorted into three categories: 1) included studies; 2) excluded studies, and 3) studies that needed more information to assess their eligibility. The authors of the studies in the third category were contacted and asked to provide the necessary information; in the impossibility of obtaining the answers, the studies were excluded from the review.

The Preferred Reporting Items for Systematic Reviews and Meta-Analysis (PRISMA) guidelines were used to guide the elaboration of the study selection flowchart.^27^

### Data extraction and management

The two main reviewers independently completed a custom data extraction form. It included the following information: study title, last name of the first author, publication year, the country where the study was conducted, study type, study population, participants’ characteristics (gender, age), tumor characteristics (size, laterality), follow-up time, treatment characteristics (therapeutic dose, cochlear dose, previous microsurgery) and audiological characteristics before and after radiosurgery (Gardner–Robertson classification; PTA).

### Assessment of the certainty of evidence and risk of bias

The certainty of the evidence was evaluated by two independent reviewers using the Grading of Recommendations, Assessment, Development, and Evaluation (GRADE), according to which three factors can increase the certainty of the evidence of observational studies: magnitude of effect, dose-response gradient, and all potential confounding factors, which increase confidence in the estimated effect. On the other hand, the certainty of the evidence can be lowered by five factors: risk of bias, inconsistency, indirectness, imprecision, and publication bias.^28^, ^29^, ^30^, ^31^, ^32^

For the risk of bias assessment, the same reviewers conducted the analysis using the Newcastle–Ottawa Quality Assessment Scale (NOS) for cohort studies.

### Data synthesis

A descriptive approach was carried out with text and tables for data synthesis. The results compilation table were made by two independent blind reviewers and contains clinically relevant information on population, sex, age, tumor size and laterality, follow-up time, previous microsurgery performance, Gardner–Robertson and PTA classifications at the beginning of the cohort, radiation therapy dose, as well as information on mean cochlear dose in both patients groups with or without hearing loss and/or the number of patients exposed to a high cochlear radiation dose that progressed to loss of hearing function.

## Results

### Study selection

In total, 333 articles were identified in the database search. 166 duplicate records were excluded. After evaluating titles and abstracts based on eligibility criteria, 122 articles were excluded. Of the 45 remaining articles, 13 did not have available full texts. 32 papers were read in full, 25 of which were excluded for the following reasons: Age group included participants <20 years old (11 articles), treatment had fractionated radiotherapy and not just single-dose radiosurgery (3 articles), study exposure was not cochlear dose (2 articles), clinical treatment was performed after radiosurgery (3 articles), included participants with anacusis (1 article), included participants with cochlear nerve schwannoma (1 article), lacked information on the eligibility criteria and it was not possible to obtain a response from the lead author (3 articles did not report whether treatment was performed before radiosurgery and 1 article did not determine the age group of the population).

**Figure 1:**
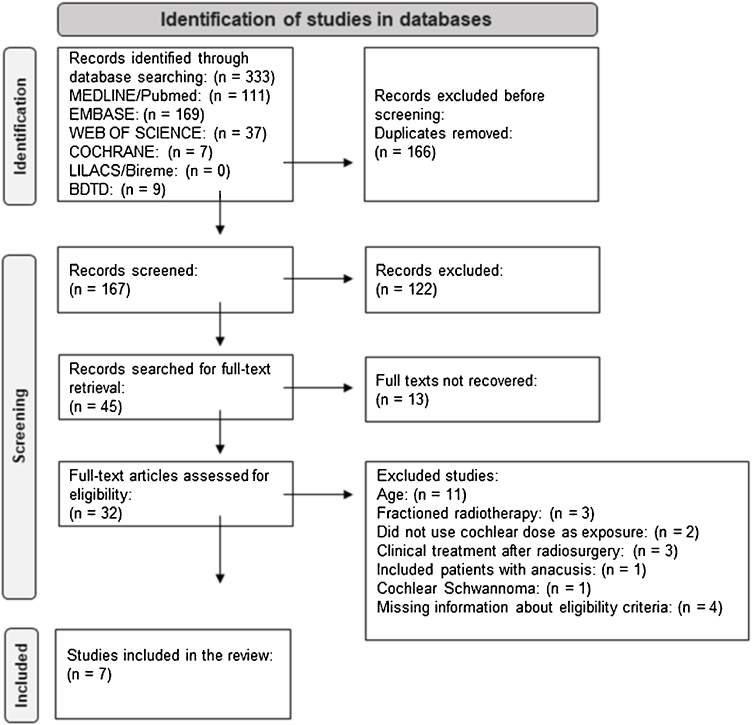
Flowchart of article selection steps following the Preferred Reporting Items for Systematic Reviews and Meta-Analysis (PRISMA), 2020. Source: created by the author using the PRISMA (2020).

Finally, seven studies were included in the review^11^, ^13^, ^14^, ^15^, ^16^, ^25^, ^33^ (Fig. 1).

### Characteristics of the studies

Table 2 shows the characteristics of the included studies published in four countries from 2005 to 2019: six retrospective cohorts and one prospective cohort. The studies included patients aged 21–87 who underwent a radiation therapy dose of 9–14 Gy, with an audiometric follow-up after radiosurgery from 0.77 to 141 months. Only two studies^16^, ^25^ included patients with non-usable hearing at the beginning of the cohort.

**Table 2:** Characteristics of studies associating cochlear radiation dose and decline in auditory function in patients with vestibular schwannoma undergoing radiosurgery.

Authors, year (country)	Type of study	Sample characteristics	Exposition	Outcomes	Results
Paek et al., 2005^11^ (Korea)	Retrospective cohort	Sample: 25 7 women and 18 men. Age: median 47 (22–65) years. Tumor size: 3.62 (0.16−9.1) cm^3^. Median follow-up: 49 (26−77) months. Previous microsurgery: 1 patient. GR classification at cohort start: 13 class I and 12 class II patients. PTA at cohort start: 28.64 (8−48) dB. GR classification at cohort end: 13 preserved GR I or II PTA at cohort end: mean 47.12 (20−80) dB. Maximum cochlear dose: mean 8.1 ± 3.1 Gy. Radiation therapy dose: mean 12 (11−14) Gy.	Maximum cochlear dose	Hearing preservation (pre- and post-radiosurgery PTA difference <20 dB). No hearing preservation (pre- and post-radiosurgery PTA difference ≥20 dB). Sample dichotomization: with or without hearing preservation *PTA: average hearing thresholds (in dB) at frequencies of 500, 1000 and 2000 Hz	Mean maximum cochlear dose in the hearing preservation group (n = 9): 9.1 ± 4.4 Gy vs. mean maximum cochlear dose in the group without hearing preservation (n = 16): 7.8 ± 2.6 Gy.
Brown et al., 2011^16^ (United States)	Retrospective cohort	Sample: 53. 31 women and 22 men. Age: mean 56 (36−87) years. Tumor size: 1.11 (0.043–4) cm^3^. Left side: 28. Right side: 25. Follow-up: mean 15.43 (0.77–36.2) months. Previous microsurgery: 1 patient. GR classification at cohort start: 31 class I and II patients. PTA at cohort start: mean 42.9 (15−88.3) dB. GR classification at cohort end: 19 preserved GR I or II PTA at cohort end: mean 54.3 (13.3−121.6) dB. Mean cochlear dose: mean 5.3 (2.5−10.5) Gy. Radiation therapy dose: median 12.5 (12−13) Gy.	Mean and maximum cochlear dose	Hearing preservation (pre- and post-radiosurgery PTA difference <20 dB). No hearing preservation (pre- and post-radiosurgery PTA difference ≥20 dB). Sample dichotomization: with or without hearing preservation *PTA: average hearing thresholds (in dB) at frequencies of 500, 1000 and 5000 Hz	Average mean cochlear dose in the hearing preservation group (n = 42): 5.1 Gy vs. average mean cochlear dose in the group without hearing preservation (n = 11): 6 Gy. Mean maximum cochlear dose in the hearing preservation group (n = 42): 11.9 Gy vs. mean maximum cochlear dose in the group without hearing preservation (n = 11): 14.1 Gy.
Yomo et al., 2012^25^ (France)	Retrospective cohort	Sample: 154 77 women and 77 men. Age: mean 54.1 (24–76) years. Tumor size: 0,73 (0.03–5.37) cm^3^. Follow-up: mean 52 (7–123) months. Previous microsurgery: 0. GR classification at cohort start: 50 class I, 55 class II, 47 class III and 2 class IV patients. PTA at cohort start: mean 40.4 ± 18.2 dB. GR classification at cohort end: 64 preserved GR I or II. PTA at cohort end: mean 55.4 ± 22.8 dB. Radiation therapy dose: mean 12.1 (9−14) Gy.	Maximum cochlear dose Sample dichotomization: ≤4 Gy vs. >4 Gy	AHDR: PTA change in dB divided by the number of years *PTA: average hearing thresholds (in dB) at frequencies of 500, 1000, 2000 and 4000 Hz	Mean AHDR in maximum cochlear dose group >4 Gy: 4.43 vs. mean AHDR in the maximum cochlear dose ≤4 Gy: 3.14.
Page et al., 2013^27^ (United States)	Prospective cohort	Sample: 40. Age: median 49 (26–80) years. Tumor size: 0.23 (0.05–4.3) cm^3^. Follow-up: mean 34.5 (6.1–57.8) months. Previous microsurgery: 1 patient. GR classification at cohort start: 28 class I and 12 class II patients. PTA at cohort start: (3–48) dB. GR classification at cohort end: 30 preserved GR I or II. Maximum cochlear dose: median 6.9 (1.6–16) Gy. Mean cochlear dose: median 2.7 (0.7–5) Gy. Radiation therapy dose: 12.5 Gy on 38 patients and 13 Gy on 2.	Mean cochlear dose Sample dichotomization: ≥3 Gy vs. <3 Gy	Usable hearing preservation (GR I or II class maintenance). Usable hearing loss (change to GR class III, IV or V).	Usable hearing loss in the mean cochlear dose group: ≥3 Gy = 41% <3 Gy = 9%
Kim et al., 2013^14^ (Korea)	Retrospective cohort	Sample: 60 37 women and 23 men. Age: mean 49.6 (21–69) years. Tumor size: 0.34 (0.03–1) cm^3^. Follow-up: mean 61.5 (36–141) months. Previous microsurgery: 1 patient. GR classification at cohort start: 35 class I and 25 class II patients. PTA at cohort start: mean 28.4 (6–50) dB. GR classification at cohort end: 34 preserved GR I or II PTA at cohort end: mean 42.1 (8–80) dB. Maximum cochlear dose: mean 8.2 (2.7−16.6) Gy. Mean cochlear dose: mean 4.2 (1.6−8.9) Gy. Radiation therapy dose: mean 12.2 (11,5−13) Gy.	Mean and maximum cochlear dose	Usable hearing preservation (GR I or II class maintenance). Usable hearing loss (change to GR class III, IV or V). Sample dichotomization: loss or preservation of usable hearing	Mean maximum cochlear dose in the usable hearing preservation group (n = 34): 7.7 Gy vs. mean maximum cochlear dose in the usable hearing loss group (n = 26): 8.8 Gy. Average mean cochlear dose in the usable hearing preservation group: 3.9 Gy vs. average mean cochlear dose in the usable hearing loss group: 4.7 Gy.
Jacob et al., 2014^13^ (United States)	Retrospective cohort	Sample: 59 32 women and 27 homens. Age: mean 58.9 (33–79) years. Left side: 27. Right side: 32. Follow-up: mean 25.2 (6–46) months. Previous microsurgery: 1 patient. GR classification at cohort start: 59 class I and II patients. PTA at cohort start: mean 32.3 (8–50) dB. GR classification at cohort end: 38 preserved GR I or II Maximum cochlear dose: mean 11.8 (3.7–19.2) Gy. Mean cochlear dose: mean 4.9 (1.9–8.3) Gy. Radiation therapy dose: 12 Gy on 38 patients and 13 Gy on 21.	Mean cochlear dose Sample dichotomization: ≥5 Gy vs. <5 Gy	Usable hearing preservation (GR I or II class maintenance). Usable hearing loss (change to GR class III, IV or V).	Usable hearing loss at 1–3 years, respectively, in the mean cochlear dose group: ≥5 Gy = 7%, 29% and 63% <5 Gy = 0%, 4% and 24%
Prabhuraj et al., 2019^15^ (India)	Retrospective cohort	Sample: 77 39 women and 38 men. Age: mean 39.46 (22–67) years. Tumor size: 4.28 (0.4–10.5) cm^3^. Follow-up: mean 30 (24–80) months. Previous microsurgery: 1 patient. GR classification at cohort start: 42 class I and 35 class II patients. PTA at cohort start: mean 28.71 (8.3–50) dB. GR classification at cohort end: 61 preserved GR I or II PTA at cohort end: mean 39.76 (5–71.6) dB. Maximum cochlear dose: mean 5.9 (3.8–10) Gy. Mean cochlear dose: mean 3.74 (2.3–6.5) Gy. Radiation therapy dose: mean 12 (11.5–14) Gy.	Mean cochlear dose Sample dichotomization: >4 Gy vs. <4 Gy	Usable hearing preservation (GR I or II class maintenance). Usable hearing loss (change to GR class III, IV or V).	Usable hearing loss in the mean cochlear dose group: >4 Gy = 7/27 <4 Gy = 9/50

cm^3^, Cubic centimeters; GR, Gardner–Robertson; PTA, Pure Tone Audiometry; dB, decibel; Gy, gray; vs., versus; Hz, hertz; AHDR, Annual Hearing Decrease Rate.

Values are presented as mean or median ± standard deviation, and in the parentheses are the minimum and maximum values, when applicable.

Source: created by the author (2022).

Regarding exposure, five studies evaluated the mean cochlear radiation dose.^13^, ^14^, ^15^, ^16^, ^33^ Of these, two additionally assessed the maximum cochlear dose.^14^, ^16^ Two studies analyzed exposure based only on maximum cochlear dose.^11^, ^25^ The sample was dichotomized in four studies, based on exposure,^13^, ^15^, ^25^, ^33^ with divergent cut-off points: mean cochlear dose of 3 Gy (1 study), mean cochlear dose of 5 Gy (1 study), mean cochlear dose of 4 Gy (1 study) and maximum cochlear dose of 4 Gy (1 study).

Four studies used the Gardner–Robertson classification for outcome assessment, considering a decline to classes III, IV or V as loss of usable hearing.^13^, ^14^, ^15^, ^33^ Two studies used the PTA difference ≥20 dB^11^, ^16^ as a criterion for the absence of hearing preservation. One study used the definition of Annual Hearing Decrease Rate (AHDR) to assess the outcome.^25^ The population was dichotomized based on the outcome of three studies, which evaluated the mean cochlear dose (mean or maximum) in groups with and without preservation of hearing function.

### Risk of bias and certainty of evidence of the included studies

The summary of the risk of bias analysis for the cohort studies included in this review is shown in Table 3.

**Table 3:** Risk of bias for cohort studies using the Newcastle–Ottawa Quality Assessment Scale (NOS), 2009–2017.

Author, year (country)	Selection				Comparability	Outcome		
	1	2	3	4	1	1	2	3
Paek, 2005 (Korea)	D	A✵	A✵	A✵	X	A✵	A✵	D
Brown, 2010 (United States)	A✵	A✵	A✵	B	X	A✵	B	D
Yomo, 2012 (France)	A✵	A✵	A✵	B	X	B✵	B	D
Baschnagel, 2013 (United States)	A✵	A✵	A✵	A✵	X	B✵	B	D
Kim, 2013 (Korea)	A✵	A✵	A✵	A✵	X	B✵	A✵	D
Jacob, 2014 (United States)	A✵	A✵	A✵	A✵	X	A✵	B	D
Prabhuraj, 2019 (India)	A✵	A✵	A✵	A✵	X	A✵	A✵	D

**Selection**
1) Representativeness of the exposed cohort
a) Truly representative of the average **VS patients treated with SRS** in the community ✵
b) Somewhat representative of the average **VS patients treated with SRS** in the community ✵
c) Selected group of users e.g. nurses, volunteers
d) No description of the derivation of the cohort
2) Selection of the non exposed cohort
a) Drawn from the same community as the exposed cohort ✵
b) Drawn from a different source
c) No description of the derivation of the non exposed cohort
3) Ascertainment of exposure
a) Secure record (e.g. surgical records) ✵
b) Structured interview ✵
c) Written self report
d) No description
4) Demonstration that outcome of interest was not present at start of study
a) Yes ✵
b) No
**Comparability**
1) Comparability of cohorts on the basis of the design or analysis
a) Study controls for **pretreatment hearing level** (select the most important factor) ✵
b) Study controls for any additional factor ✵

**Outcome**
1) Assessment of outcome
a) Independent blind assessment ✵
b) Record linkage ✵
c) Self report
d) No description
2) Was follow-up long enough for outcomes to occur
a) Yes (>**12 months**) ✵
b) No
3) Adequacy of follow up of cohorts
a) Complete follow up — all subjects accounted for ✵
b) Subjects lost to follow up unlikely to introduce bias – small number lost – ≥**90%** follow up, or description provided of those lost ✵
c) Follow up rate <**90%** and no description of those lost
d) No statement

VS, vestibular schwannoma; SRS, Stereotactic Radiosurgery.

Source: created by the author based on the Newcastle–Ottawa Quality Assessment Scale (NOS) (2022).

Most studies involved representative populations, except for one,^11^ which did not clearly describe the cohort selection. The unexposed cohort was drawn from the same community as the exposed in all studies, and all exposure records were safe, coming from the radiosurgery programming software. Not usable hearing was present at the beginning of the cohort in only two studies.^16^, ^25^

It was not possible to judge the comparability between exposed and unexposed cohorts in any of the studies.

Four studies extracted the outcome from secure medical records with detailed audiometry.^11^, ^13^, ^15^, ^16^ The others used databases to store and access information. Three studies performed an audiometric follow-up of more than 12 months^11^, ^14^, ^15^ and none of the studies described the adequacy of the follow-up of the cohorts.

It was only possible to adequately assess the certainty of evidence with GRADE for useful hearing loss measured by the Gardner–Robertson classification at exposure to a mean cochlear dose of >4 Gy (Table 4). Only one study^15^ carried out this evaluation, with a total number of participants of 77, with 50 from the control group and 27 from the group exposed to high cochlear dose. Follow-up ranged from 24 to 80 months, and the relative risk for hearing loss was 1.44 (95% CI 0.6–3.44).

**Table 4:** Summary of results: mean cochlear dose of >4 Gy compared to mean cochlear dose of <4 Gy in adults with vestibular schwannoma undergoing radiosurgery.

Patient or population: adults with vestibular schwannoma undergoing radiosurgery						
Intervention: mean cochlear dose >4 Gy						
Comparison: mean cochlear dose <4 Gy						
Outcomes	Potential absolute effects* (95% CI)		Relative effect (95% CI)	Nº of participants (studies)	Certainty of the evidence (GRADE)	Comments
	Risk with mean cochlear dose <4 Gy	Risk with mean cochlear dose >4 Gy				
**Loss of usable hearing with:** Gardner–Robertson classification (decline from class I or II to III, IV or V) follow-up: 24–80 months	18–100	26 per 100 (11–62)	RR 1.44 (0.60–3.44)	77 (1 observational study)	⨁◯◯◯ Very low^a^, ^b^, ^c^	

CI, confidence interval; RR, risk ratio.

GRADE Working Group grades of evidence.

High certainty: we are very confident that the true effect lies close to that of the estimate of the effect.

Moderate certainty: we are moderately confident in the effect estimate: the true effect is likely to be close to the estimate of the effect, but there is a possibility that it is substantially different.

Low certainty: our confidence in the effect estimate is limited: the true effect may be substantially different from the estimate of the effect.

Very low certainty: we have very little confidence in the effect estimate: the true effect is likely to be substantially different from the estimate of effect.

* The risk in the intervention group (and its 95% CI) is based on the assumed risk in the comparison group and the relative effect of the intervention (and its 95% CI).

a Risk of bias: serious.

b Inconsistency: serious.

c Imprecision: very serious.

The risk of bias was serious due to the lack of adjustment for confounders. The outcome was measured in a single study, so inconsistency assessment was not possible, which was therefore categorized as severe. There was no indirect evidence or publication bias, but there was imprecision, which lowered the certainty of the evidence at two levels due to the low number of events and the confidence interval that crosses the line of statistical significance. None of the domains raised the level of evidence, and the certainty of evidence was very low.

### Summary of results

There was no standardization regarding measuring exposure and outcome in the studies included in this review. Only the maximum cochlear dose was evaluated in two studies. Two studies assessed maximum cochlear dose only,^11^, ^25^ three assessed mean cochlear dose only^13^, ^15^, ^33^ and two evaluated both.^16^, ^33^ Additionally, only four studies defined a cut-off point for the characterization of “high dose”^13^, ^15^, ^25^, ^33^ and there was a divergence between the adopted cut-off points (average cochlear doses of 3–5 Gy and a maximum cochlear dose of 5 Gy). Regarding hearing assessment, one study used the AHDR^25^ as a criterion for hearing decline, and two used changes in the PTA testing.^11^, ^16^ The others used the drop in the Gardner–Robertson class from I or II to III, IV or V.

The studies did not provide sufficient information about the exposed and unexposed groups. Only one study^15^ reported the absolute number of participants in each cohort and the patients who showed the hearing decline outcome. For this study, the cut-off point adopted for the definition of “high dose” was a mean cochlear dose of 4 Gy, and the relative risk for the outcome was 1.44 (95% CI 0.60‒3.44). Therefore, it was not possible to perform a meta-analysis.

## Discussion

In the present review, it was not possible to determine a consensual cut-off point for the definition of high cochlear dose. Despite using different definitions, the included studies showed a relationship between the use of higher mean cochlear doses and a decline in auditory function. However, it was not possible to estimate measures of effect size and certainty of evidence due to insufficient data presented in the articles.

The dose measurement method varied between studies. The maximum radiation dose that reaches a single point in the cochlea, the average radiation dose that reaches the cochlear volume, and the dose that reaches the cochlear modiolus have been described as significant substitutes for calculating the cochlear dose when monitoring the risk of radiosurgery therapy complications. Each of these measurements represents a distinct value and cannot be easily interchanged, as there is no constant correlation between them. In addition, the size and shape of the tumor, the distance from the tumor to the cochlea, and the radiosurgical planning itself are implicated in the way and intensity with which the radiation reaches the cochlear volume and need to be considered when comparing such measurements.^34^ But this was not possible in the studies included in the review.

Mean cochlear dose and/or maximum cochlear dose were the main ways of measuring the cochlear dose. The mean cochlear dose, which corresponds to the mean amount of radiation reaching the entire cochlear volume, was measured in five studies.^13^, ^14^, ^15^, ^16^, ^33^ Four of these defined a value for “high dose”, ranging from 3 to 5 Gy.^13^, ^14^, ^15^, ^33^ And in all of them, there was an association between high cochlear dose and a decline in auditory function.

The maximum cochlear dose, which corresponds to the maximum radiation dose that reaches a single point in the cochlea, was analyzed by four studies.^11^, ^14^, ^16^, ^25^ Of these, only one defined a high dose value, established from the 4 Gy cut-off point.^35^ This same study did not refer to the mean cochlear dose. Of the studies included in the review, two did not determine any value for high cochlear dose.^11^, ^16^

The criterion to define the decline in hearing function also differed between studies. The 1988 Gardner–Robertson classification, which considers tone thresholds and speech discrimination, defines usable hearing thresholds as less than 50 dB and discrimination greater than 50%. Below these values, hearing is considered not usable. Subjects with near-normal hearing before radiosurgery may present a hearing loss of 20 dB and remain classified as usable hearing by that classification. Also, patients with borderline hearing with a decline of less than 20 dB may be classified as having hearing loss, changing to Gardner–Robertson class III. Furthermore, patients with hearing thresholds that are better than expected for their discrimination percentage may have their hearing overestimated in the assessment exclusively guided by the PTA. For this reason, the authors of the present review considered it clinically inappropriate to combine the two classifications in the same analysis.

The risk of bias analysis was performed using the NOS, and the factors common to all studies that would denote a higher risk of bias were the lack of comparability between cohorts and the absence of follow-up adequacy definition. The review studies evaluated several prognostic factors related to the hearing outcome in the patients in question, and there was no characterization of the cohorts for each exposure. Regarding the follow-up period, this was determined before conducting the study and was part of the eligibility criteria since six of the seven articles included were retrospective cohorts.^11^, ^13^, ^14^, ^15^, ^16^, ^25^ It is understood, therefore, that subjects with a very short follow-up period were excluded from the study and not characterized as being lost to follow-up.

Due to the lack of exposure standardization, the different ways of assessing the outcome, and the presentation of incomplete data, the certainty of evidence assessment consisted of only a single study,^15^ with a relative risk for decline in auditory function of 1.44 (95% CI 0.6‒3.44). The risk of bias, inconsistency, and imprecision resulted in very low certainty of evidence.

### Limitations

As limitations of this review, it is essential to note that only observational studies were included, which usually leads to less certainty of evidence. However, this limitation stems from the authors’ proposal to assess exposure, so that is the most appropriate type of study found for that matter. Furthermore, every systematic review is susceptible to the methodological limitations of the original articles. The incomplete presentation of data from the cohorts is highlighted, which leads to publication bias since the data were hidden, especially in the analyses that did not find statistical significance, with small samples and without favorable results. It may also represent publication bias, the 13 records excluded for being only abstracts, without published article. Such abstracts did not have the necessary data for inclusion in this review.

In addition, the cochlear dose measurement methodology, as well as the definition of high dose, and the way of measuring auditory function were quite heterogeneous, making it impossible to carry out a meta-analysis.

The authors are unaware of a previous systematic review that evaluated cochlear radiation dose and decline in auditory function in patients with vestibular schwannoma undergoing radiosurgery. One strength of the study is the methodological rigor with which the review was conducted, in compliance with the MOOSE guidelines. The definition of strict eligibility criteria, taking care of defining clinically relevant outcomes based on objective audiometry data, reflects the effort to seek relevant results for patients in medical practice.

It is suggested that future studies adopt the mean cochlear dose to measure exposure, as it reflects the radiation that reaches the entire cochlear volume and not just a specific point in the cochlea. Determining a cut-off point from which there is a significant decline in hearing function is crucial for the clinical management of these patients and can potentially optimize the audiological prognosis in radiosurgery. Evaluating the outcome should also be standardized, and the Gardner–Robertson classification is considered a clinically relevant outcome, as it considers tonal thresholds and speech recognition.

## Conclusion

It was not possible to determine the cut-off point for the definition of high cochlear radiation dose that would constitute a risk factor for the decline of hearing function in adults with vestibular schwannoma submitted to radiosurgery.

## Authors’ contributions

The work covers different areas of expertise. The authors who conducted independently and concomitantly all stages of the review, Gabriela S.T. Cavalcanti and Camilla Maria G.A. Lucena, received guidance from the authors Silvio Caldas and Mariana C. Leal in the area of otology, Lílian F. Muniz and Leonardo G.A. Venâncio in the area of audiology, João Gabriel R. Gomes in the area of neurosurgery and radiosurgery and from Andrea Lemos and Eduarda C. Moretti in the methodological conduction of the systematic review, so that all contributed in an essential way with the final result of the article.

## Conflicts of interest

The authors declare no conflicts of interest.
